# Platelet dysfunction in hypercholesterolemia mice, two Alzheimer’s disease mouse models and in human patients with Alzheimer’s disease

**DOI:** 10.1007/s10522-015-9580-1

**Published:** 2015-05-07

**Authors:** Barbara Plagg, Josef Marksteiner, Kathrin M. Kniewallner, Christian Humpel

**Affiliations:** Laboratory of Psychiatry and Experimental Alzheimer’s Research, Department of Psychiatry and Psychotherapy, Medical University of Innsbruck, Anichstr. 35, 6020 Innsbruck, Austria; Department of Psychiatry and Psychotherapy A, Landeskrankenhaus Hall, Hall in Tirol, Austria

**Keywords:** Alzheimer’s disease, Platelets, Biomarker, Amyloid-precursor protein, Epidermal growth factor, Serotonin

## Abstract

Alzheimer’s disease (AD) is a severe neurodegenerative disorder characterized mainly by accumulation of amyloid-β plaques and neurofibrillary tangles, synaptic and neuronal loss. Blood platelets contain the neurotransmitter serotonin and amyloid-precursor protein (APP), and may thus be useful as a peripheral biomarker for AD. The aim of the present study was to functionally characterize platelets by FACS, to examine alterations in APP expression and secretion, and to measure serotonin levels in hypercholesterolemia mice with AD-like pathology and in two AD mouse models, the triple transgenic AD model (3xTg) and the APP overexpressing AD model with the Swedish–Dutch–Iowa mutations (APP_SweDI). These data are supplemented with epidermal growth factor (EGF) levels and compared with changes observed in platelets of patients with AD. We observed decreased platelet APP isoforms in 3xTg mice and patients with AD when analysed by means of Western blot. In patients, a significant increase of APP levels was observed when assessed by ELISA. Secreted APPβ proved to be altered amongst all three animal models of AD at different time points and in human patients with AD. Serotonin levels were only reduced in 7 and 14 month old 3xTg mice. Moreover, we found significantly lower EGF levels in human AD patients and could thereby reproduce previous findings. Taken together, our data confirm that platelets are dysfunctional in AD, however, results from AD animal models do not coincide in all aspects, and markedly differ when compared to AD patients. We support previous data that APP, as well as EGF, could become putative biomarkers for diagnosing AD in human platelets.

## Introduction

Alzheimer’s disease (AD) is a progressive neurodegenerative disease that gradually leads to severe cognitive deterioration and premature death. The main neuropathological hallmarks of AD concurrently include accumulation of amyloid-β (Aβ) plaques, neurofibrillary tangles (NFT), inflammation and glial responses, synaptic and neuronal loss and vascular alterations. In addition, reduced acetylcholine concentrations as well as a severe decrease of monoamine transmitters serotonin, dopamine and norepinephrine are observed in patients with AD (Trillo et al. 2013).

AD is classified into clinically indistinguishable early-onset familiar AD (EOAD) and late-onset sporadic AD (LOAD). While EOAD accounts for approximately 1–5 % of all cases and is associated with autosomal dominantly inherited mutations with a high penetrance, LOAD accounts for >95 % of all AD cases and turned out to be a complex heterogeneous disease. Effectively, the diagnosis of possible or probable AD requires according to the current National Institute of Neurological and Communicative Disorders and Stroke and the AD and Related Disorders Association (NINCDS–ADRDA) criteria both the presence of cognitive impairment established by clinical and neuropsychological examination and the absence of other diseases capable of producing a dementia syndrome, while definite AD diagnosis still requires post-mortem evaluation of plaques and NFTs in brain tissue (McKhann et al. 1984; Dubois et al. 2007). Thus, the diagnosis of AD within the clinical routine is based on a time consuming combination of psychological testing, imaging and the analysis of three well-established biomarkers (amyloid-β_42_ (Aβ_42_), total tau and phospho-tau-181) in cerebrospinal fluid (CSF) (Blennow 2005; Blennow et al. 2010; Zetterberg et al. 2010; Tapiola et al. 2009; Dubois et al. 2007). The increasing rate of AD cases not only stresses the establishment of efficient therapeutic intervention, but also the identification of economic and reliable biomarkers in blood, urine, or saliva since CSF collection is an invasive procedure and an expensive analysis (Humpel 2011). We and others extensively searched for biomarkers in plasma/serum (Blennow et al. 2010; Ray et al. 2007; Song et al. 2009; Hu et al. 2012; Chiu et al. 2013), peripheral blood mononuclear cells (Hu et al. 2014; Kassner et al. 2008), monocytes (Hochstrasser et al. 2012a; Naert and Rivest 2013; Shad et al. 2013; Bradshaw et al. 2013) or platelets (Adolfsson et al. 1980; Di Luca et al. 1996; Borroni et al. 2010; Casoli et al. 2010; Marksteiner and Humpel 2013).

Platelets can be considered a valid peripheral model to study metabolic mechanisms related to AD: these anuclear blood cells share several biochemical and homeostatic functions with neural cells such as accumulation and release of neurotransmitters like serotonin, glutamate and dopamine, and expression of membrane-bound components as receptors and enzymes (El Haouari and Rosado 2009). Also, human platelets are known to be the most important source for more than 90 % of the circulating APP (Li et al. 1994; Padovani et al. 2001) and store Aβ (especially Aβ_40_) in their granules, which is released upon stimulation by physiological agonists like thrombin, collagen or calcium ionophores (Li et al. 1999; Bush et al. 1990; Kokjohn et al. 2011; Skovronsky et al. 2001). Recently, also tau protein, the major component of NFTs seen in AD, has been identified in platelet proteome (Neumann et al. 2011). Moreover, platelets are known to play an important role in a variety of cardiovascular, psychosomatic, psychiatric and neurodegenerative diseases. On these grounds, platelets have been a promising target within the search for peripheral biomarkers in AD.

Up to now, several studies have reported significantly lower platelet APP ratio (i.e. the ratio between the upper 130 kDa and the lower 106–110 kDa isoforms) in AD patients correlating positively with cognitive decline (Di Luca et al. 1996, 1998; Borroni et al. 2004; Tang et al. 2006; Zainaghi et al. 2012). Discrepant results regarding platelet serotonin concentration, processing and uptake have been published: increased and decreased, as well as unchanged serotonin metabolism were reported, precluding a final evaluation on platelet serotonin status in AD (Tukiainen et al. 1981; Andersson et al. 1991; Prokšelj et al. 2014; Mimica et al. 2008; Muck-Seler et al. 2009; Kumar et al. 1995; Koren et al. 1993; Arora et al. 1991). Decreased platelets EGF, as well as increased sAPPβ levels in patients with AD and MCI were previously reported by our group (Hochstrasser et al. 2012b; Marksteiner and Humpel 2013). Other platelet components such as activation, membrane fluidity or enzymatic activity have been investigated in patients with AD, inconsistent results have, however, to date impeded the establishment of a valid platelet-derived biomarker.

Mutant mouse models exhibiting traits such as plaque and tangle pathology, vascular aberrations and cognitive decline similar to those seen in AD have been widely used in AD research. However, transgenic AD mouse models represent more a familiar form of AD and reflect only partly the entire AD pathology. The APP_SweDI mouse model, harbouring the Swedish K670N/M671L and vasculotropic Dutch/Iowa E693Q/D694N mutations, is a well-established model showing severe Aβ plaque-like depositions starting at 6 months of age, cognitive decline and vascular pathology including cerebral amyloid angiopathy (CAA) but no tau pathology (Davis et al. 2004). The triple-transgenic model (3xTg) however, develops a neuropathological phenotype including besides synaptic dysfunction both Aβ plaques and tangle pathology at late stage (Oddo et al. 2003). Along with genetic interventions, weak traits of sporadic AD and visible cognitive decline are seen in different animals and AD mouse models with hypercholesterolemia (Sparks et al. 1994; Ullrich et al. 2010). While cholesterol diet triggers in wildtype control mice a weak decline in cognition most pronounced at 7 months, it elicits only very weak AD-like pathology (Hohsfield et al. 2014). Thus, the three mouse models may partly reflect AD pathology and are a useful tool to study AD-related processes. While recently platelet activation and thrombus formation in APP23 mice was investigated (Jarre et al. 2014), to the best of our knowledge platelet APP, sAPPβ and serotonin have not been studied in hypercholesterolemia and the two AD (APP_SweDI and 3xTg) mouse models.

The aim of the present study is to extensively characterize AD-related alterations of platelet APP processing and secretion of sAPPβ, and to measure serotonin levels in three different mouse models: the hypercholesterolemia mouse model, the triple transgenic model displaying Aβ plaques and tau pathology at late stage, and the APP_SweDI mouse model showing Aβ plaques at an early stage, but no tau pathology. These data will be supplemented and compared with changes observed in platelets from patients with AD.

## Materials and methods

### Animals

Wildtype (WT, 129/C57BL6 or C57BL/6N), triple-transgenic AD (3xTg-AD, B6; 129-*Psen1*^*tm1Mpm*^Tg(APPSwe, tauB301L)1Lfa/J) and transgenic APP_SweDI (Tg-SweDI; expressing amyloid precursor protein (APP) harboring the Swedish K670N/M671L, Dutch E693Q, and Iowa D694N mutations; C57BL/6-Tg(Thy1-APPSwDutIowa) BWevn/Mmjax) mice were purchased from The Jackson Laboratory and housed at the Medical University of Innsbruck animal facility providing open access to food and water under 12/12 h light–dark cycles. All animals were genotyped according to standardized methods. At 2 months of age, wildtype mice began to receive either a normal diet or a well-established 5 % cholesterol diet as described by us in detail (Ullrich et al. 2010; Hohsfield et al. 2014). The cholesterol containing nutrition was composed of: 450 g/kg cornstarch, 140 g/kg casein, 155 g/kg maltodextrin, 100 g/kg sucrose, 40 g/kg soybean oil, 50 g/kg fiber, 35 g/kg mineral mix, 1.8 g/kg l-cystine, 1.4 g/kg choline chloride, 0.0008 g/kg butylhydroxytoluol, 10 g/kg vitamin mix (without folic acid), 1 g/kg chocolate aroma, 0.002 g/kg folic acid and 50 g/kg cholesterol for animals on the cholesterol diet (ssniff special diet, Soest, Germany). All animal experiments were approved by the Austrian Ministry of Science and Research (BMWF-66.011/0044-II/3b/2011 and BMWF-66.011/0059-II/3b/2011) and conformed to the Austrian guidelines on animal welfare and experimentation. All possible steps were taken to reduce suffering and the number of animals used during the experiment.

### Patients

Cognitively healthy subjects and patients suffering from AD were recruited at the *Landeskrankenhaus* Hall/Tirol, Austria. Thirty healthy persons (10 men and 20 women, aged 77 ± 1 years) and 43 patients with AD (18 men and 25 women, aged 80 ± 1 years) were included in this study. Both groups were assessed by the same diagnostic procedure. Diagnosis of AD was established by a structured routine process including clinical assessment, extensive neuropsychological tests (including mini-mental state examination, MMSE) and neuroimaging (magnetic resonance imaging, MRI) to exclude other brain pathologies. Probable AD was diagnosed according to the current NINCDS–ARDRA criteria and confirmed for all participating patients. A general blood examination was part of the routine diagnostic procedure. Exclusion criteria for healthy subjects, MCI and AD patients included (1) another primary neurological or mental disorder, (2) any kind of metabolic decompensation or any sign of peripheral inflammation (e.g. rheumatic disease), (3) long-term alcohol or drug abuse, (4) or any current, clinically significant cardiovascular disease. The study was approved by the ethics committee of Medical University of Innsbruck. All subjects and/or their caregivers enrolled in the study gave their informed consent.

### Isolation of mouse platelets and processing

Mouse platelets were isolated as reported previously (Marksteiner and Humpel 2013). Briefly, mice were anaesthetized by an intraperitoneal injection of sodium thiopental (12.5 mg/ml, 1 ml; Sandoz, Kundl, Austria). Blood drawn directly from the heart was immediately collected in ethylenediaminetetraacetate (EDTA) tubes (S-monovettes, Sarstedt, Germany), gently mixed and instantly centrifuged at 100×*g* for 10 min in order to part platelet rich plasma (PRP). PRP (upper phase) was collected and prostaglandine 2 (PGI2, 500 nM, Sigma, Vienna, Austria) added according to the obtained quantity of PRP (19 µl/ml) to avoid platelet activation during processing. Next, PRP was partitioned into two portions and then centrifuged at 400×*g* for 10 min to isolate platelets. An aliquot was dissolved in Tyrode buffer (136 mM NaCl, 2.7 mM KCl, 12 mM NaHCO_3_, 0.42 mM NaH_2_PO_4_, 1 mM MgSo_4_, 5 mM glucose, pH 6.5) and immediately analysed (6 µl) by FACS. The rest was dissolved in 100 µl 1 × secretase buffer (20 mM sodium acetate, 0.2 % Triton, pH 4.5), sonicated with a Branson sonifier (10 strokes each 10 s on ice), then centrifuged at 16,000×*g* for 10 min and the supernatant was collected, total protein was measured using the Bradford protein assay and the rest was frozen at −80° C until use.

### Isolation of human platelets and processing

Human platelets were isolated as previously described (Marksteiner and Humpel 2013). In short, 10 ml blood was collected in EDTA tubes during normal clinical routine, centrifuged at 250×*g* for 10 min at RT to collect platelet-rich plasma (PRP). PRP was centrifuged for 10 min at RT (2300×*g*) and the pellet was dissolved in 1 ml Tyrode’s buffer and PGI2 was added in order to inhibit platelet activation. Next, the dissolved pellet was partitioned into three portions with each 330 µl and then centrifuged at 2300×*g* for 10 min.

#### Sample A-human

The pellet was resuspended in 150 µl secretase buffer (20 mM sodiumacetate, 0.2 % Triton, pH 4.5), sonicated with a Branson sonifier (10 strokes each 10 s on ice), then centrifuged at 14,000×*g* for 10 min and total protein determined by Bradford assay. The rest was frozen at −80 °C until use.

#### Sample B-human

The pellet was resuspended in 100 µl phosphate buffered saline (PBS) with a protease inhibitor cocktail (Sigma), sonicated with a Branson sonifier (10 strokes each 10 s on ice, 125 W/cm^2^, 140 µm amplitude, 100 %), and centrifuged at 14,000×*g* for 10 min. The supernatant was collected, again the total protein determined and frozen at −80 °C.

#### Sample C-human

To the platelet pellet 100 µl Tyrode’s buffer was added and analysed by FACS. The rest was incubated with 2 mM CaCl_2_ at 37 °C for 60 min. Afterwards, the platelets were centrifuged at 1900×*g* for 10 min and the supernatant was frozen at −80 °C, while the pellet was dissolved in 100 µl of 0.4 N NaOH (1 h, 45 °C) and again analysed for total protein content.

### FACS analysis

Immediately after isolation, 2 µl mouse platelets were incubated with antibodies against IgG1-FITC (Milteny Biotec, Bergisch Gladbach, Germany, order no.: 130-098-847, 1:25) or CD62P-FITC (BD Biosciences, Heidelberg, Germany, Cat: 561923, 1:25). Human platelets were incubated with antibodies against FITC Mouse IgG1 (BD Biosciences, Cat: 555748, 1:25) or FITC Mouse Anti-Human CD62P (BD Biosciences, Cat: 555523, 1:25). Subsequently, 46 µl of FACs buffer (2 mM EDTA, 0.5 % FCS ad 100 ml PBS, pH 7.1) was added and the samples incubated for 30 min at 4 °C in dark. To study apoptosis, 2 µl of platelets were incubated with 5 µl Annexin V-FITC (Milteny Biotec, Order no.: 130-097-928) in 100 µl Annexin V Binding Buffer (Miltenyi Biotec, 20x Stock Solution) at RT for 30 min in dark. Immediately before analysis, 2 µl of 7-AAD (Miltenyi Biotec) was added. All samples were centrifuged at 300*g* for 10 min and the pellets were resuspended in 100 µl of FACs flow (BD FACSFlow). FACs analysis was instantly performed with a BD FACScan.

### Serotonin analysis by HPLC

Platelet serotonin was measured by high-performance liquid chromotography (HPLC) as previously described in detail by us (Ullrich et al. 2010). Thus, 20 µl thawed supernatants or extracts were injected on the HPLC column. As the mobile phase a mixture of 0.05 M trichloric acid (Merck, Darmstadt, Germany), 0.26 mM EDTA, 1.36 mM NaCl, 1.81 mM heptanesulfonic acid and 15 % acetonitrile in HPLC water was used. The components of the samples were separated on a reversed-phase C18 Nucleosil column (Bartelt, Graz, Austria) at a flow rate of 1 ml/min. For the detection of serotonin an electrochemical detector Antec II was used. The amount of serotonin in the samples was calculated based on the peak height at the serotonin-specific retention time (7.8 min).

### Western blot analysis

In order to analyse the expression of platelet APP, Western blot analysis was performed. Five µg acidic platelet extracts (neutralized with PBS pH 9.5) were denatured (10 min 70 °C) and loaded onto 4–12 % Bis–Tris polyacrylamide gel (Invitrogen Life Tech, Darmstadt, Germany) and separated by electrophoresis for 60 min at 200 V. Consecutively, samples were electrotransferred to nylon PVDF Immobilon-PSQ membranes (Millipore, Vienna, Austria) at 30 V for 90 min with 20 % methanol transfer buffer (Invitrogen). Protein detection was performed with Western Breeze Chemiluminescent System (Invitrogen). Thus, membranes were blocked for 30 min with blocking solution at RT on the shaker, then incubated with the primary antibody (Anti-Amyloid beta precursor protein antibody [Y188], abcam, Cambridge, UK, ab32136) overnight at 4 °C. Following, blots were washed and incubated with anti-rabbit antibodies for 30 min at RT, again washed and incubated with CDP-Star chemiluminescent substrate solution (Invitrogen). Imaging was performed with a cooled CCD camera SearchLight camera. In order to quantify the protein amounts in each lane and ensure that there has been an even transfer from gel to membrane, a loading control with αMouse IgG was performed.

### APP ELISA

Platelet APP was measured using the APP ELISA (Invitrogen KHB0051, code 310314) kit according to the instruction manual. Briefly, 50 µl of neutralized extracts were mixed with 50 µl of standard diluent and 2 µl of sample reducing agent (NuPage, Invitrogen) and denatured (10 min 70 °C) and then put on ice. Next, 100 µl of each sample/standard was added to the pre-coated wells, incubated overnight at 4 °C, then washed, 100 µl of biotinylated antibody was added and again incubated at RT for 1 h. Afterwards, 100 µl STREP-HRP was added, incubated at RT for 30 min, washed, 100 µl of chromogen was added and incubated at RT in dark for 30 min. Last, 100 µl of stop solution was added and measured in the Zenyth ELISA reader (at 450 nm). The APP values were calculated according to the standard curve.

### sAPPβ ELISA

Secreted APPβ was analysed using the sAPPβ-w (highly sensitive) Assay Kit—IBL (code 27732, Immuno-Biological Laboratories, Hamburg, Germany) according to the manufacturer description. In brief, 12.5 µg of the acidic platelet extracts were incubated in 50 µl secretase buffer (pH 4.5) for 2.5 h at 37 °C in order to elicit the secretion process. Subsequently, 50 µl of EIA buffer (1 % BSA, 0.05 % Tween20 in PBS) was added and a total of 100 µl for each sample/standard were incubated in the prepared wells overnight at 4 °C, then washed, 100 µl of labelled antibody was added, incubated for 30 min at 4 °C, again washed, 100 µl of chromogen was added and incubated at RT in dark for 30 min. All incubation steps were carried out on a shaker. Successively, 100 µl stop solution was added and the luminescent signal was detected with an ELISA reader Zenyth 3100 and the absorbance was measured at 450 nm. The samples were calculated according to a standard curve.

### Human EGF ELISA

Platelet EGF levels and EGF release after incubation were measured using human EGF ELISA kit (RayBiotech, ELH-EGF-001, THP Vienna, Austria). In short, PBS extracted samples (5 µl) or supernatants (30 µl) were added in 100 µl diluent into pre-coated wells, covered and incubated for 2.5 h at RT with gentle shaking. Next, the solution was washed and 100 µl of biotinylated antibody was added and again incubated for 1 h at RT. Afterwards, the solution was again discarded and washed and 100 µl of Streptavidin solution was added. After 45 min the solution was discarded and washed and 100 µl of TMP One-Step Substrate Reagent was added and incubated for 30 min. Last, 50 µl of Stop Solution was added and with an ELISA reader Zenyth 3100 the signal was detected (450 nm). The EGF values were calculated according to the standard curve.

### Statistical analysis

Statistical analysis was performed by one Way ANOVA with a subsequent Fisher LSD posthoc test where p < 0.05 was significant.

## Results

### Wildtype control mice

**Fig. 1:**
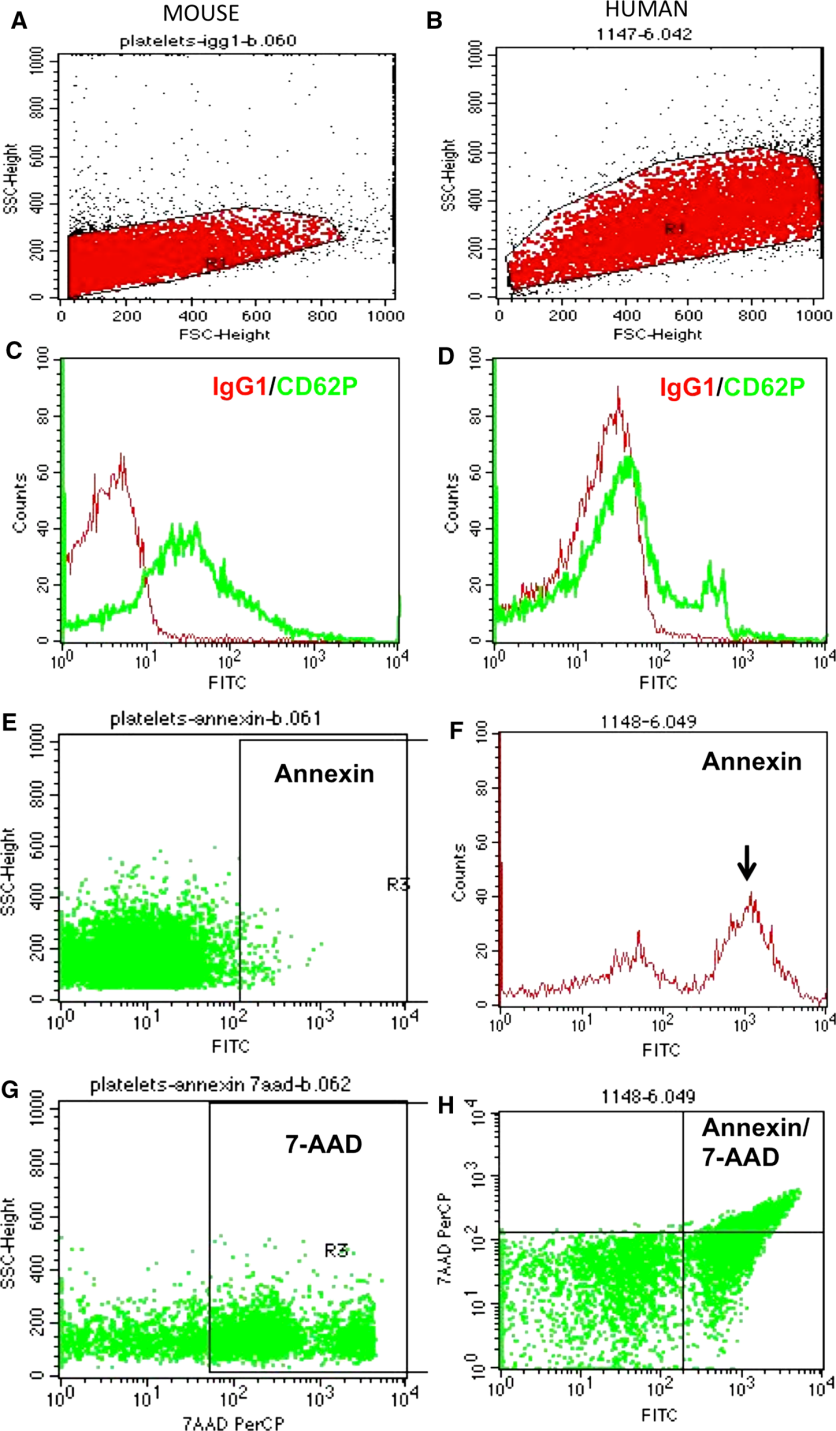
Flow cytometry (FACS) analysis in mouse (**a**, **c**, **e**, **g**) and human (**b**, **d**, **f**, **h**) platelets shows a common population of cells in forward and sideward scatter (**a**, **b**) and markedly expressed CD62P in mouse (**c**, *green*) but less in human (**d**, *green*) platelets, compared to an IgG control (*red*). Apoptosis was assessed using Annexin IV staining (**e**, **f**), showing examples of no apoptosis in mice (**e**) or increased apoptosis in humans (**f**, *arrow*). Necrosis was measured using 7-amino actinomycin D (7-AAD) in a mouse example (**g**) or co-expressed in apoptotic human platelets (**h**). (Color figure online)

**Fig. 2:**
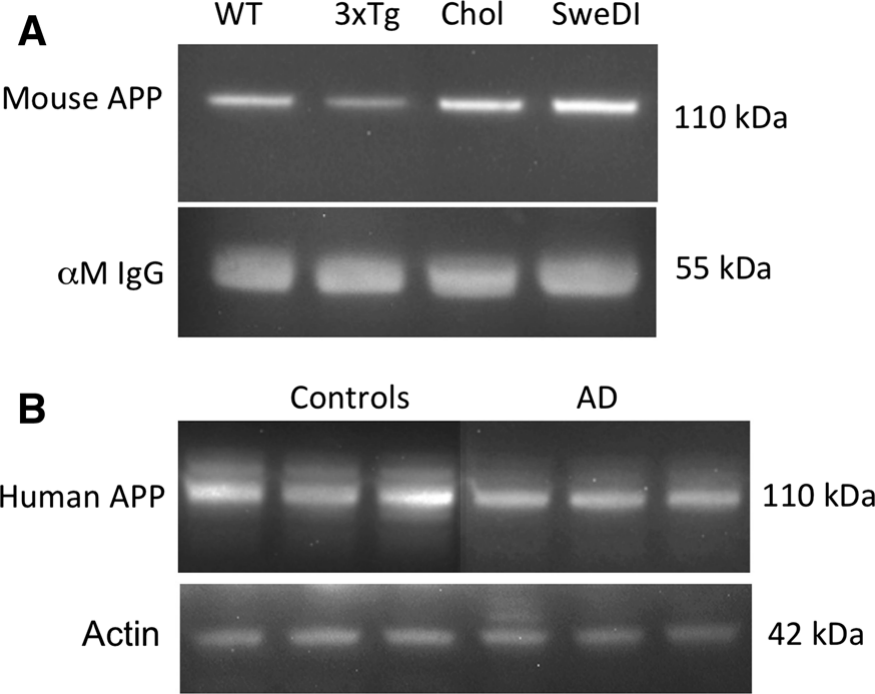
Western blot analysis of amyloid precursor protein (APP) immunoreactivity in mouse (**a**) or human (**b**) platelets. In mice, one single APP band was seen at approx. 110 kDa in wildtype (WT), triple transgenic (3xTg), cholesterol (Chol) or APP_SweDI (SweDI) mice. Loading control was performed using anti-mouse IgG showing a band at 55 kDa (**a**). In humans, 2 bands were visible one larger at approximately 130 kDa and a smaller at approximately 110/106 kDa (**b**). The larger 130 kDa band declined in Alzheimer’s disease patients (AD) compared to controls (**b**). Actin at 42 kDa served as a loading control

Mouse platelets from wildtype mice (C57BL/6N) were isolated and characterized using FACS analysis, displaying a common population and marked CD62P expression (Fig. 1). Apoptotic and necrotic cell death was very low in young animals (<0.5 %) and slightly increased in older mice (<9 %). Serotonin levels were around 60 ng/mg protein and increased in older animals (Tables 1, 2, 3). APP like immunoreactivity was detectable in Western Blot as a single band at approximately 110 kDa (Fig. 2), but did not markedly change during aging. APP as measured by ELISA was found to be approximately 2 ng/mg protein which increased in older animals (Table 3). The release of sAPPβ was approximately 3 ng/ml × 12.5 µg × 150 min in WT mice.

**Table 1:** Hypercholesterolemia mouse model

Months	sAPPβ	APP 110 kDa	Serotonin
7			
WT	3780 ± 330 (5)-	99 ± 8 (5)-	59 ± 11 (5)-
WT Chol	3600 ± 330 (5) ns	80 ± 8 (5) ns	62 ± 11 (4) ns
14			
WT	3809 ± 332 (5)	146 ± 16 (5)-	70 ± 8 (5)-
WT Chol	2097 ± 909 (4) ns	180 ± 29 (5) ns	58 ± 15 (5) ns
20			
WT	2077 ± 464 (4)	76 ± 18 (6)	640 ± 127 (5)-
WT Chol	868 ± 396 (4) p = 0.09	105 ± 33 (5) ns	529 ± 163 (5) ns

Wildtype mice (WT) were fed with or without 5 % cholesterol (Chol). At the age of 7, 14 or 20 months platelets from WT or WT-cholesterol animals were isolated and processed for secreted amyloid precursor protein beta (sAPPβ) release, APP and serotonin. Values are given as mean ± SEM pg/ml × 25 µg × 150 min for sAPPβ, optical density (OD) for APP and ng/mg protein for serotonin. *Values in parenthesis* give the number of animals. Statistical analysis was performed by One Way ANOVA with a subsequent Fisher-LSD posthoc test

*ns* not significant

**Table 2:** Triple transgenic (3xTg) Alzheimer’s disease mouse model

Months	sAPPβ	APP 110 kDa	Serotonin
7			
WT	3780 ± 330 (5)-	99 ± 8 (5)-	59 ± 11 (5)-
TG	2360 ± 740 (5)*	52 ± 16 (5)*	20 ± 2 (5)**
14			
WT	3809 ± 332 (5)	146 ± 16 (5)-	70 ± 8 (5)-
TG	1899 ± 552 (5)*	49 ± 21 (5)**	332 ± 165 (4)*
20			
WT	2077 ± 464 (4)	76 ± 18 (6)	640 ± 127 (5)-
TG	580 ± 325 (4)*	55 ± 19 (5) ns	365 ± 123 (5) ns

At the age of 7, 14 or 20 months platelets from wildtype (WT) or 3xTg mice were isolated and processed for secreted amyloid precursor protein beta (sAPPβ) release, APP and serotonin. Values are given as mean ± SEM pg/ml × 25 µg × 150 min for sAPPβ, optical density (OD) for APP and ng/mg protein for serotonin. *Values in parenthesis* give the number of animals. Statistical analysis was performed by One Way ANOVA with a subsequent Fisher-LSD posthoc test (* p < 0.05; ** p < 0.01)

*ns* not significant

**Table 3:** APP_SweDI Alzheimer’s disease mouse model

	6 months		12 months	
	WT	TG	WT	TG
Cell population (%)	66 ± 14 (4)	87 ± 2 (6) ns	77 ± 2 (6)	83 ± 2 (6) ns
CD620P (%)	39 ± 12 (4)	42 ± 11 (6) ns	53 ± 5 (6)	76 ± 3 (6)**
Annexin V (%)	0.5 ± 0.25 (4)	18 ± 5 (6)*	8.4 ± 1.8 (6)	32 ± 8 (6)*
Annexin/7-AAD (%)	0.09 ± 0.02 (4)	2.7 ± 0.7 (6)*	2.4 ± 0.6 (6)	12 ± 4 (6)*
APP WB 110 kDa (OD)	134 ± 47 (7)	129 ± 30 (7) ns	183 ± 18 (6)	140 ± 21 (6) ns
APP (ng/mg protein)	2.1 ± 0.8 (6)	1.4 ± 0.3 (6) ns	9.1 ± 3.1 (5)	12.8 ± 6.7 (6) ns
sAPPβ (pg/ml × 25 µg × 150 min)	1399 ± 254 (4)	755 ± 88 (6)*	606 ± 144 (6)	403 ± 144 (6) ns
Serotonin (ng/mg protein)	177 ± 78 (5)	223 ± 89 (6) ns	291 ± 99 (6)	397 ± 96 (6) ns

At the age of 6 or 12 months platelets from wildtype (WT) or APP_SweDI (TG) mice were isolated and processed for FACS analysis, secreted amyloid precursor protein beta (sAPPβ) release, APP (Western blot and ELISA) and serotonin. Values are given as mean ± SEM; *values in parenthesis* give the number of animals. Statistical analysis was performed by One Way ANOVA with a subsequent Fisher-LSD posthoc test (* p < 0.05; ** p < 0.01)

*ns* not significant, *OD* optical density, *WB* Western blot

### Hypercholesterolemia mouse model

A cholesterol diet (5 %) for 7, 14 or 20 months did not significantly affect the platelet APP markers and serotonin (Table 1). Only a tendency for decreased sAPPβ release was observed in 20 month old hypercholesterolemic mice (Table 1).

### Triple transgenic AD mouse model

APP 110 kDa as measured by Western Blot significantly decreased in 7, and 14 months but not in 20 months old 3xTg mice (Table 2; Fig. 2). A significant reduction of sAPPβ was observed at 7 and 14 months and more pronounced in 20 months old mice (Table 2). In the 3xTg mice there was a significant reduction of serotonin levels at 7 months, which was less noticeable in 14 months old animals and remained stable at 20 months (Table 2).

### APP_SweDI AD mouse model

In this AD mouse model platelets showed markedly enhanced apoptosis and necrosis as determined by FACS analysis (Table 3; Fig. 1). Also, significantly increased CD62P positive cells in 12 months old transgenic animals were seen (Table 3). APP 110 kDa did not change at any timepoint compared to controls (Fig. 2). The release of sAPPβ was significantly reduced at 6 months, but not at 12 months (Table 3). Serotonin levels were not significantly changed at any point in time (Table 3).

### Human samples

In the human cohort, 43 patients with AD and 30 age-matched and cognitively healthy persons were included. As expected, the MMSE scores were significantly lower in the AD-group (18 ± 1) compared to healthy subjects (29 ± 0.3) (Table 4). AD patients were significantly older (80 ± 1 years) compared to the healthy volunteers (77 ± 1 years).

**Table 4:** Human platelets

	Controls	AD
Age (years)	77 ± 1 (30)	80 ± 1 (43)*
Sex (m/f)	10/20	18/25 ns
MMSE	29 ± 0.3 (30)	18 ± 1 (43)***
Cell population (%)	50 ± 2 (30)	56 ± 3 (43) ns
CD62P (%)	30 ± 3 (30)	26 ± 3 (43) ns
Annexin (%)	29 ± 3 (30)	33 ± 4 (43) ns
Annexin/7ADD (%)	15 ± 2 (30)	18 ± 2 (43) ns
APP 130 kDa (OD)^a^	75 ± 10 (15)	38 ± 7 (25)*
APP (110/106 kDa) (OD)^a^	71 ± 9 (15)	42 ± 8 (25) p = 0.08
APP (ng/mg protein)	2.6 ± 0.3 (30)	3.3 ± 0.2 (43)*
sAPPβ (pg/ml × 25 µg × 150 min)^c^	519 ± 50 (33)	1548 ± 211 (68)***
sAPPβ (pg/ml × 25 µg × 150 min)	402 ± 102 (26)	924 ± 174 (41)*
Serotonin (ng/mg protein)^b^	565 ± 79 (11)	529 ± 88 (22) ns
Serotonin (ng/ml × mg × h)	22 ± 7 (30)	16 ± 5 (39) ns
EGF (pg/mg protein)	365 ± 56 (25)	170 ± 66 (36)*
EGF (pg/ml × mg × h)	66 ± 7 (25)	87 ± 9 (36) p = 0.09

Platelets were isolated from healthy controls or Alzheimer’s disease patients (AD) and processed for FACS analysis, secreted amyloid precursor protein beta (sAPPβ) release, APP (Western Blot and ELISA), serotonin or EGF. Values are given as mean ± SEM; *values in parenthesis* give the number of patients/controls. Statistical analysis was performed by Student’s *t* test (* p < 0.05; *** p < 0.001)

*ns* not significant, *OD* optical density, *MMSE*, mini mental state examination

^a^Taken from Ehrlich et al. (2013) Platelets 24:26–36

^b^Taken from Hochstrasser et al. (2012b) Curr. Alzheimer Res. 9:982–989

^c^Taken from Marksteiner and Humpel (2013) Curr. Neurovasc. Res. 10:297–303 using a Covance ELISA

Platelets were efficiently isolated (50 ± 2 % IgG1 positive cells in healthy controls and 56 ± 3 % in patients with AD; Table 4; Fig. 1). FACS analysis revealed a moderate staining for CD62P (30 ± 3 for controls and 26 ± 3 % for AD patients) (Table 4; Fig. 1). Also, moderate necrosis and apoptosis as visualized by Annexin and 7AAD was observed (Table 4; Fig. 1).

A reduction in the optical density of the 130 kDa isoform and a tendency towards a reduction of the 110/106 kDa isoform was found in our present human samples (Fig. 2; Table 4). However, the total platelet APP levels as measured by ELISA were significantly increased (Table 4). Using a novel IBL assay kit, significantly decreased (×1.8) secreted APPβ in AD patients was found (Table 4). Although the measured levels were lower as compared to a previous used Covance ELISA system, the ratio of this decrease (×1.5) remained similar (Table 4). Finally, a significant decrease in platelet EGF levels was found with a tendency towards increased EGF release (Table 4).

## Discussion

Platelets modulate multiple cellular processes including angiogenesis and inflammation (Gawaz et al. 2005; Patzelt and Langer 2012) and are involved in a variety of pathologic conditions where they actively aggravate vascular changes as e.g. artherosclerotic events (Gawaz et al. 2005). Since it is known that AD-pathology is accompanied by both inflammatory and vascular damaging processes, it seems likely that platelets actively contribute to the progression of AD. Moreover, besides neurotransmitters, platelets contain proteins such as APP and produce beta-amyloid and secretases, making them an interesting target to study peripheral changes in AD as they may be useful biomarkers of the disease (Rainesalo et al. 2005; Chen et al. 1995; Li et al. 1999; Evin et al. 2003). In the present study we assessed platelet APP, sAPPβ and serotonin in three different mouse models and human patients with AD; additionally, we evaluated platelet EGF levels in our human cohort, in order to evaluate whether these components are altered in AD and whether platelets are useful as peripheral biomarkers in AD.

### Platelet population, CD62P and apoptosis

Activated platelets undergo a rapid shape change and redistribute several proteins from their granules to their surface. In order to trigger the acquisition of activated platelets with the flow cytometer, surface-bound P-selectin was identified with a FITC-conjugated monoclonal antibody CD62P, a platelet-specific antigen. A negative control (IgG1) was incorporated in the protocol and the obtained values substracted from the CD62P positive events. CD62P is located on the inner membrane of α-granules and released on the outside upon activation where it acts as a receptor and is thus a platelet activation/degranulation marker. Regarding platelet activation as quantified by CD62P, contradicting results have been published: an elevated degree of platelet activation (Sevush et al. 1998), as well as no difference of membrane-attached P-selectin without agonist stimulation in AD was reported (Järemo et al. 2013). Also, increased P-selectin expression at baseline was found to significantly associate with faster cognitive decline (Stellos et al. 2010). In the present study we did not find significantly increased membrane-attached P-selectin expression in human patients with AD and young APP_SweDI mice. However, we found a significant increase in CD62P binding in 12 month old APP_SweDI mice. This was accompanied by enhanced cell death as visualized with 7-aminoactinomycin D and significantly higher apoptoptis as seen with Annexin V. This suggests that platelets, besides increased activation, are also more sensitive to cell death in the AD mouse model. In contrast, human patients did not show altered platelet populations compared to healthy controls suggesting that mouse platelets are more vulnerable or that the AD mice do not fully display the human AD pathology.

### Serotonin

 Once released, platelet-derived serotonin modulates platelet aggregation at sites of vascular injury. As it has been demonstrated that activated platelets aggregate at sites of vascular Aβ promoting CAA by inducing platelet thrombus formation (Roher et al. 2009; Gowert et al. 2014), it is tempting to assume serotonin alterations in AD.

In the present study we observed significantly reduced platelet serotonin concentration in 3xTg mice at early stage. This can be due to both, enhanced platelet activity or decreased serotonin uptake in these transgenic mice. However, we did not see any alterations of serotonin concentrations in dietary hypercholesterolemia mouse models, APP_SweDI animals and human patients with AD. Thus, while some studies report reduced platelet serotonin content in hypercholesterolemia subjects and animal models (Smith and Betteridge 1997; Ogawa et al. 2000), we cannot report similar findings in our hypercholesterolemia mouse population.

 Previous studies including ours failed to demonstrate altered serotonin processing in AD (Tukiainen et al. 1981; Andersson et al. 1991; Hochstrasser et al. 2012b), while a number of studies reported impaired uptake of platelet serotonin (Koren et al. 1993; Inestrosa et al. 1993) and significantly lower platelet serotonin concentration (Prokšelj et al. 2014; Mimica et al. 2008; Muck-Seler et al. 2009). These findings stand in contrast to other studies, postulating increased serotonin concentration (Kumar et al. 1995, Milovanovic et al. 2014). The discrepancy of these results may arise from different AD stages, as well as from diurnal and seasonal variations of serotonin (Muck-Seler et al. 2009; Meyerson et al. 1989). We conclude that platelet serotonin levels fluctuate and have a high variability, and are therefore not suited as a reliable biomarker for AD.

### Amyloid-precursor protein in platelets

Platelets are an important peripheral source of APP, and three major isoforms (770, 751 and 695) are inserted in the membrane of resting platelets (Gardella et al. 1990; Bush et al. 1990; Di Luca et al. 2000). While APP695 lacks the KPI and OX-2 domains and is mainly expressed in neuronal cells, platelets preferably express the two KPI containing APP isoforms 770 and 751 (Tanzi et al. 1988; Golde et al. 1990; Mönning et al. 1992; Li et al. 1994). Additionally, platelets are also equipped with α-, β-, and γ-secretases, thus being able to generate different Aβ fragments (Colciaghi et al. 2004). Up to date, the physiological role of APP and its metabolites in platelets is not fully understood.

In several studies including ours, platelet APP ratio between the larger and lower isoforms of AD patients is significantly lower compared to healthy control subjects and correlate with disease severity (Di Luca et al. 1996, 1998, 2000; Borroni et al. 2004; Padovani et al. 2001; Baskin et al. 2000; Rosenberg et al. 1997; Zainaghi et al. 2012; Ehrlich et al. 2013). However, APP has not yet been established as a diagnostic biomarker for early AD (Zainaghi et al. 2012; Srisawat et al. 2013) because of its low diagnostic accuracy and methodological problems. In the present study, we confirm that platelet APP ratio is decreased, but we included in addition to Western Blot a quantitative analysis of the present cohort where we found the opposite trend. Moreover, we show for the first time that APP 110 kDa isoform is reduced in the triple transgenic AD mouse model. This suggests that the 3xTg AD mouse model shares some similarities with the human AD pathology and indeed this model displays plaques as well as Tau pathology. Accordingly, the APP 110 kDa isoform was unchanged in the hypercholesterolemia and APP_SweDI mouse models. In contrast to the findings that APP isoforms are decreased, our present study shows that the quantitative ELISA analysis of total APP reveals rather an increase of APP in human samples. While this seems to be a mismatch, it is likely that the ELISA system recognizes several forms of APP including secreted APP forms. Thus, the Western Blot is a more accurate method to differentiate between the APP isoforms. The development of an ELISA system highly specific for the 130 kDa isoform may be of potential interest to increase reproducibility and sensitivity.

### Secreted APPβ

Secreted APPβ is released as an extracellular cleavage product after the first step within the amyloidogenic processing of APP. While the function of sAPPβ is not entirely clear, it has been proposed that secreted APP has neuroprotective and synapse promoting activities in the brain (Bandyopadhyay et al. 2007). Both, sAPPβ and sAPPα were moreover associated with decreased cell adhesion and increased axon elongation in vitro (Chasseigneaux et al. 2011). Our group has recently investigated both platelet sAPPβ and sAPPα levels, finding that sAPPβ but not sAPPα is markedly increased in MCI and AD patients (Marksteiner and Humpel 2013). In the present study we confirm a significantly increased sAPPβ release in AD patients. However, the formerly used Covance ELISA resulted in slightly higher levels of sAPPβ compared to the IBL ELISA used in this study. The observed increase of sAPPβ release is an indicator for enhanced APP cleavage in platelets of patients with AD. In contrast to the increase of sAPPβ release in AD patients, we found significantly decreased sAPPβ release in two transgenic mouse models (3xTg and APP_SweDI) and additionally a trend towards reduced sAPPβ release in old (20 months) hypercholesterolemia mice. This controversy may be again due to the different pathological framework of mouse models and human patients. Altogether, while finding an opposed trend in mouse and humans, we were able to replicate our previous findings in human patients with a different ELISA assay. Thus, platelet sAPPβ release may represent therefore an interesting target worth further investigation.

### Epidermal growth factor in platelets

Growth factors play an important role in proliferation, differentiation and growth of numerous cell types. Platelets contain several growth factors, amongst others EGF, vascular endothelial growth factor or platelet-derived growth factor. Along with other proteins, EGF was found to be increased in plasma of patients with AD (Marksteiner et al. 2011; Björkqvist et al. 2012). As previously reported by our group, platelet EGF was significantly reduced in patients with AD (Hochstrasser et al. 2012b). In the present study we confirm that platelet EGF is reduced in AD patients. This reduction as seen in our studies stands in concordance with a described increase of plasma EGF levels, possibly caused by a continuous release of EGF by platelets under AD-conditions.

### Comparison mouse and human platelets

This study shows that platelets undergo distinct changes in mouse models of AD and patients with AD compared to either wild type animals or cognitively healthy subjects. The results for AD mouse models are consistent for a decrease in APPβ secretion, which was found in both transgenic animal models. However, this alteration was inversely pronounced in human samples, where we observed a significant increase in secreted APPβ in patients with AD. The reduction of the optical density of APP (130 kDa) in humans is likewise observed in the 110 kDa isoform of 3xTg animals. An inconsistency was observed for serotonin levels and serotonin release with significant changes only in the triple transgenic model. A decrease in platelet EGF levels in human patients with AD confirms our previous findings. The overall results indicate that changes in AD are not limited to the central nervous system, but co-occur in peripheral blood cells such as platelets. More specifically, platelet EGF levels are altered in patients with AD and platelet APP expression and secretion is altered in AD patients with supporting evidence from AD mouse models. Both proteins, APP and EGF are worth further investigation as they may be useful peripheral biomarkers for AD. Hypercholesterolemia did not significantly trigger platelet changes in AD mouse models, supporting the hypothesis that the observed changes are AD-specific, while a cholesterol diet alone does not alter platelet serotonin levels, APP expression or the secretion of APPβ.

However, there are noticeable differences among the three mouse models and human patients with AD. The differences among the models and AD patients may reflect pathophysiological and neuropathological differences. Although we assessed platelets of highly valid transgenic animal models of AD and hypercholesterolemia mice with comparable cognitive deficits, none of the currently available models recapitulates all pathological conditions of the human AD brain and they appear limited in mirroring sensitive peripheral changes as seen in the present study. Moreover, while mice represent an excellent model for studying platelet biology in vivo because murine platelets share properties with human platelets, they differ per se in some ultrastructural characteristics. We also found that platelets of a transgenic AD mouse population are generally more susceptible for apoptosis and necrosis compared to human platelets. While the significant tendency of reduced APP (130 kDa) as seen by Western blot in human patients with AD was supported by findings in our 3xTg group (110 kDa), the secretion of APPβ in transgenic animals is reversely pronounced as compared to humans. The discrepancy found in the secretion of APPβ by platelets demonstrates that murine peripheral APP is diversely processed in response to pathological conditions that are only of limited dimension compared to those seen in humans. Since APP secretion is closely linked to platelet activation, the observed difference may possibly be due to altered platelet reactivity in mouse models as seen by enhanced CD62P expression and cell death. Because platelet APP expression and processing seem overall affected in early and late AD, this protein represents a highly interesting target worth further investigation within the search for peripheral biomarkers in human subjects. Likewise, the reduction of platelet EGF levels may be a promising marker for AD-related changes and is in need of further evaluation.

### Limitations of the study including methodological problems

(1) The cross-sectional design of this experiment precludes the long-term and time-dependent variation of the different platelet components. The biological effects of all measured biomarkers may vary with different stages of diseases and at different points of time. (2) Due to low sample volumes (1 ml EDTA blood from mice), EGF and FACS analysis could not be performed in the mouse models compared to humans (10 ml EDTA blood). (3) The number of human participants included in this study is relatively low. (4) In this study, only AD patients were included. Further studies are needed to understand, whether the observed alterations are AD-specific in terms of their ability to distinguish AD from clinically similar dementia forms. (5) Platelets are sensitive cells and require careful handling during preparation. After decades of study, there is still a general need to establish concurrent methods for the different processing steps of platelets because the wide variation of reported protocols for platelet preparation leads to different biological responses and incomparable data. Several variables (such as e.g. anticoagulants in collection tubes, time of processing, etc.) in the platelet preparation process need to be standardized to minimize artificial stimulation during blood collection and sample handling.

## Conclusion

Biomarkers may have an immense scientific and clinical value through the whole progression of a disease. Our data support the hypothesis that platelets are dysfunctional in AD and AD-like conditions, however, to date no AD-specific platelet biomarker has been clinically established. The inconsistencies of intra- and inter-person variability may account for this problem. We further show that transgenic mice do not fully display the human AD pathology also in respect of platelet alterations. Altogether, we believe that platelet biomarkers may become very useful in diagnosing early and late AD.

## References

[CR1] Adolfsson R, Gottfries CG, Oreland L, Wiberg A, Winblad B (1980). Increased activity of brain and platelet monoamine oxidase in dementia of Alzheimer type. Life Sci.

[CR2] Andersson A, Adolfsson R, Eriksson K, Marcusson J (1991). Platelet [3H]paroxetine binding to 5-HT uptake sites in Alzheimer’s disease. Neurobiol Aging.

[CR3] Arora RC, Emery OB, Meltzer HY (1991). Serotonin uptake in the blood platelets of Alzheimer’s disease patients. Neurology.

[CR4] Bandyopadhyay S, Goldstein LE, Lahiri DK, Rogers JT (2007). Role of the APP non-amyloidogenic signaling pathway and targeting alpha-secretase as an alternative drug target for treatment of Alzheimer’s disease. Curr Med Chem.

[CR5] Baskin F, Rosenberg RN, Iyer L, Hynan L, Cullum CM (2000). Platelet APP isoform ratios correlate with declining cognition in AD. Neurology.

[CR7] Björkqvist M, Ohlsson M, Minthon L, Hansson O (2012). Evaluation of a previously suggested plasma biomarker panel to identify Alzheimer’s disease. PLoS One.

[CR8] Blennow K (2005). CSF biomarkers for Alzheimer’s disease: use in early diagnosis and evaluation of drug treatment. Expert Rev Mol Diagn.

[CR9] Blennow K, Hampel H, Weiner M, Zetterberg H (2010). Cerebrospinal fluid and plasma biomarkers in Alzheimer disease. Nat Rev Neurol.

[CR10] Borroni B, Colciaghi F, Archetti S, Marcello E, Caimi L, Di Luca M, Padovani A (2004). Predicting cognitive decline in Alzheimer disease. Role of platelet amyloid precursor protein. Alzheimer Dis Assoc Dis.

[CR11] Borroni B, Agosti C, Marcello E, Di Luca M, Padovani A (2010). Blood cell markers in Alzheimer disease: amyloid precursor protein form ratio in platelets. Exp Gerontol.

[CR12] Bradshaw EM, Chibnik LB, Keenan BT, Ottoboni L, Raj T, Tang A, Rosenkrantz LL, Imboywa S, Lee M, Von Korff A, Morris MC, Evans DA, Johnson K, Sperling RA, Schneider JA, Bennett DA, De Jager PL, Alzheimer Disease Neuroimaging Initiative (2013). CD33 Alzheimer’s disease locus: altered monocyte function and amyloid biology. Nat Neurosci.

[CR13] Bush AI, Martins RN, Rumble B, Moir R, Fuller S, Milward E, Currie J, Ames D, Weidemann A, Fischer P (1990). The amyloid precursor protein of Alzheimer’s disease is released by human platelets. J Biol Chem.

[CR14] Casoli T, Di Stefano G, Balietti M, Solazzi M, Giorgetti B, Fattoretti P (2010). Peripheral inflammatory biomarkers of Alzheimer’s disease: the role of platelets. Biogerontology.

[CR15] Chasseigneaux S, Dinc L, Rose C, Chabret C, Coulpier F, Topilko P, Mauger G, Allinquant B (2011). Secreted amyloid precursor protein β and secreted amyloid precursor protein α induce axon outgrowth in vitro through Egr1 signaling pathway. PLoS One.

[CR16] Chen M, Inestrosa NC, Ross GS, Fernandez HL (1995). Platelets are the primary source of amyloid beta-peptide in human blood. Biochem Biophy Res Commun.

[CR17] Chiu MJ, Yang SY, Horng HE, Yang CC, Chen TF, Chieh JJ, Chen HH, Chen TC, Ho CS, Chang SF, Liu HC, Hong CY, Yang HC (2013). Combined plasma biomarkers for diagnosing mild cognition impairment and Alzheimer’s disease. ACS Chem Neurosci.

[CR18] Colciaghi F, Marcello E, Borroni B, Zimmermann M, Caltagirone C, Cattabeni F, Padovani A, Di Luca M (2004). Platelet APP, ADAM 10 and BACE alterations in the early stages of Alzheimer disease. Neurology.

[CR19] Davis J, Xu F, Deane R, Romanov G, Previti ML, Zeigler K, Zlokovic BV, Van Nostrand WE (2004). Early-onset and robust cerebral microvascular accumulation of amyloid beta-protein in transgenic mice expressing low levels of a vasculotropic Dutch/Iowa mutant form of amyloid beta-protein precursor. J Biol Chem.

[CR20] Di Luca M, Pastorino L, Cattabeni F, Zanardi R, Scarone S, Racagni G, Smeraldi E, Perez J (1996). Abnormal pattern of platelet APP isoforms in Alzheimer disease and down syndrome. Arch Neurol.

[CR21] Di Luca M, Pastorino L, Bianchetti A, Perez J, Vignolo LA, Lenzi GL, Trabucchi M, Cattabeni F, Padovani A (1998). Differential level of platelet amyloid beta precursor protein isoforms: an early marker for Alzheimer disease. Arch Neurol.

[CR22] Di Luca M, Colciaghi F, Pastorino L, Borroni B, Padovani A, Cattabeni F (2000). Platelets as a peripheral district where to study pathogenetic mechanisms of alzheimer disease: the case of amyloid precursor protein. Eur J Pharmacol.

[CR23] Dubois B, Feldman HH, Jacova C, Dekosky ST, Barberger-Gateau P, Cummings J, Delacourte A, Galasko D, Gauthier S, Jicha G, Meguro K, O’brien J, Pasquier F, Robert P, Rossor M, Salloway S, Stern Y, Visser PJ, Scheltens P (2007). Research criteria for the diagnosis of Alzheimer’s disease: revising the NINCDS-ADRDA criteria. Lancet Neurol.

[CR24] Ehrlich D, Hochstrasser T, Humpel C (2013). Effects of oxidative stress on amyloid precursor protein processing in rat and human platelets. Platelets.

[CR25] El Haouari M, Rosado JA (2009). Platelet function in hypertension. Blood Cells Mol Dis.

[CR26] Evin G, Zhu A, Holsinger RM, Masters CL, Li QX (2003). Proteolytic processing of the Alzheimer’s disease amyloid precursor protein in brain and platelets. J Neurosci Res.

[CR27] Gardella JE, Ghiso J, Gorgone GA, Marratta D, Kaplan AP, Frangione B, Gorevic PD (1990). Intact Alzheimer amyloid precursor protein (APP) is present in platelet membranes and is encoded by platelet mRNA. Biochem Biophys Res Commun.

[CR28] Gawaz M, Langer H, May AE (2005). Platelets in inflammation and atherogenesis. J Clin Investig.

[CR29] Golde TE, Estus S, Usiak M, Younkin LH, Younkin SG (1990). Expression of beta amyloid protein precursor mRNAs: recognition of a novel alternatively spliced form and quantitation in Alzheimer’s disease using PCR. Neuron.

[CR30] Gowert NS, Donner L, Chatterjee M, Eisele YS, Towhid ST, Münzer P, Walker B, Ogorek I, Borst O, Grandoch M, Schaller M, Fischer JW, Gawaz M, Weggen S, Lang F, Jucker M, Elvers M (2014). Blood platelets in the progression of Alzheimer’s disease. PLoS One.

[CR31] Hochstrasser T, Marksteiner J, Humpel C (2012). Telomere length is age-dependent and reduced in monocytes of Alzheimer patients. Exp Gerontol.

[CR32] Hochstrasser T, Ehrlich D, Marksteiner J, Sperner-Unterweger B, Humpel C (2012). Matrix metalloproteinase-2 and epidermal growth factor are decreased in platelets of Alzheimer patients. Curr Alzheimer Res.

[CR33] Hohsfield LA, Ehrlich D, Humpel C (2014). Intravenous infusion of nerve growth factor-secreting monocytes supports the survival of cholinergic neurons in the nucleus basalis of Meynert in hypercholesterolemia brown-Norway rats. J Neurosci Res.

[CR34] Hu WT, Holtzman DM, Fagan AM, Shaw LM, Perrin R, Arnold SE, Grossman M, Xiong C, Craig-Schapiro R, Clark CM, Pickering E, Kuhn M, Chen Y, Van Deerlin VM, McCluskey L, Elman L, Karlawish J, Chen-Plotkin A, Hurtig HI, Siderowf A, Swenson F, Lee VM, Morris JC, Trojanowski JQ, Soares H (2012). Alzheimer’s disease neuroimaging initiative. Plasma multianalyte profiling in mild cognitive impairment and Alzheimer disease. Neurology.

[CR35] Hu N, Tan MS, Sun L, Jiang T, Wang YL, Tan L, Zhang W, Yu JT, Tan L (2014). Decreased expression of CD33 in peripheral mononuclear cells of Alzheimer’s disease patients. Neurosci Lett.

[CR36] Humpel C (2011). Identifying and validating biomarkers for Alzheimer’s disease. Trends Biotechnol.

[CR37] Inestrosa NC, Alarcón R, Arriagada J, Donoso A, Alvarez J (1993). Platelet of Alzheimer patients: increased counts and subnormal uptake and accumulation of [^14^C]5-hydroxytryptamine. Neurosci Lett.

[CR38] Järemo P, Milovanovic M, Buller C, Nilsson S, Winblad B (2013). P-selectin paradox and dementia of the Alzheimer type: circulating P-selectin is increased but platelet-bound P-selectin after agonist provocation is compromised. Scand J Clin Lab Investig.

[CR39] Jarre A, Gowert NS, Donner L, Münzer P, Klier M, Borst O, Schaller M, Lang F, Korth C, Elvers M (2014). Pre-activated blood platelets and a pro-thrombotic phenotype in APP23 mice modeling Alzheimer’s disease. Cell Signal.

[CR40] Kassner SS, Bonaterra GA, Kaiser E, Hildebrandt W, Metz J, Schröder J, Kinscherf R (2008). Novel systemic markers for patients with Alzheimer disease?—A pilot study. Curr Alzheimer Res.

[CR41] Kokjohn TA, Van Vickle GD, Maarouf CL, Kalback WM, Hunter JM, Daugs ID, Luehrs DC, Lopez J, Brune D, Sue LI, Beach TG, Castaño EM, Roher AE (2011). Chemical characterization of pro-inflammatory amyloid-beta peptides in human atherosclerotic lesions and platelets. Biochim Biophys Acta.

[CR42] Koren P, Diver-Haber A, Adunsky A, Rabinowitz M, Hershkowitz M (1993). Uptake of serotonin into platelets of senile dementia of the Alzheimer type patients. J Gerontol.

[CR43] Kumar AM, Sevush S, Kumar M, Ruiz J, Eisdorfer C (1995). Peripheral serotonin in Alzheimer’s disease. Neuropsychobiology.

[CR44] Li QX, Berndt MC, Bush AI, Rumble B, Mackenzie I, Friedhuber A, Beyreuther K, Masters CL (1994). Membrane-associated forms of the beta A4 amyloid protein precursor of Alzheimer’s disease in human platelet and brain: surface expression on the activated human platelet. Blood.

[CR45] Li QX, Fuller SJ, Beyreuther K, Masters CL (1999). The amyloid precursor protein of Alzheimer disease in human brain and blood. J Leukoc Biol.

[CR46] Marksteiner J, Humpel C (2013). Platelet-derived secreted amyloid-precursor protein-β as a marker for diagnosing Alzheimer’s disease. Curr Neurovasc Res.

[CR47] Marksteiner J, Kemmler G, Weiss EM, Knaus G, Ullrich C, Mechtcheriakov S, Oberbauer H, Auffinger S, Hinterhölzl J, Hinterhuber H, Humpel C (2011). Five out of 16 plasma signaling proteins are enhanced in plasma of patients with mild cognitive impairment and Alzheimer’s disease. Neurobiol Aging.

[CR48] McKhann G, Drachman D, Folstein M, Katzman R, Price D, Stadlan EM (1984). Clinical diagnosis of Alzheimer’s disease: report of the NINCDS-ADRDA Work Group under the auspices of Department of Health and Human Services Task Force on Alzheimer’s Disease. Neurology.

[CR49] Meyerson LR, Strano R, Ocheret D (1989). Diurnal concordance of human platelet serotonin content and plasma alpha-1-acid glycoprotein concentration. Pharmacol Biochem Behav.

[CR50] Milovanovic M, Eriksson K, Winblad B, Nilsson S, Lindahl TL, Post C, Järemo P (2014). Alzheimer and platelets: low-density platelet populations reveal increased serotonin content in Alzheimer type dementia. Clin Biochem.

[CR51] Mimica N, Mück-Seler D, Pivac N, Mustapić M, Dezeljin M, Stipcević T, Presecki P, Radonić E, Folnegović-Smalc V (2008). Platelet serotonin and monoamine oxidase in Alzheimer’s disease with psychotic features. Coll Antropol.

[CR52] Mönning U, König G, Banati RB, Mechler H, Czech C, Gehrmann J, Schreiter-Gasser U, Masters CL, Beyreuther K (1992). Alzheimer beta A4-amyloid protein precursor in immunocompetent cells. J Biol Chem.

[CR53] Muck-Seler D, Presecki P, Mimica N, Mustapic M, Pivac N, Babic A, Nedic G, Folnegovic-Smalc V (2009). Platelet serotonin concentration and monoamine oxidase type B activity in female patients in early, middle and late phase of Alzheimer’s disease. Prog Neuropsychopharmacol Biol Psychiatry.

[CR54] Naert G, Rivest S (2013). A deficiency in CCR2+ monocytes: the hidden side of Alzheimer’s disease. J Mol Cell Biol.

[CR55] Neumann K, Farías G, Slachevsky A, Perez P, Maccioni RB (2011). Human platelets tau: a potential peripheral marker for Alzheimer’s disease. J Alzheimers Dis.

[CR56] Oddo S, Caccamo A, Shepherd JD, Murphy MP, Golde TE, Kayed R, Metherate R, Mattson MP, Akbari Y, LaFerla FM (2003). Triple-transgenic model of Alzheimer’s disease with plaques and tangles: intracellular Abeta and synaptic dysfunction. Neuron.

[CR57] Ogawa T, Sugidachi A, Asai F, Koike H (2000). Reduced platelet serotonin content in rabbits with dietary hypercholesterolemia. Blood Coagul Fibrinolysis.

[CR58] Padovani A, Pastorino L, Borroni B, Colciaghi F, Rozzini L, Monastero R, Perez J, Pettenati C, Mussi M, Parrinello G, Cottini E, Lenzi GL, Trabucchi M, Cattabeni F, Di Luca M (2001). Amyloid precursor protein in platelets: a peripheral marker for the diagnosis of sporadic AD. Neurology.

[CR59] Patzelt J, Langer HF (2012). Platelets in angiogenesis. Curr Vasc Pharmacol.

[CR61] Prokšelj T, Jerin A, Muck-Seler D, Kogoj A (2014). Decreased platelet serotonin concentration in Alzheimer’s disease with involuntary emotional expression disorder. Neurosci Lett.

[CR62] Rainesalo S, Keränen T, Saransaari P, Honkaniemi J (2005). GABA and glutamate transporters are expressed in human platelets. Mol Brain Res.

[CR63] Ray S, Britschgi M, Herbert C, Takeda-Uchimura Y, Boxer A, Blennow K, Friedman LF, Galasko DR, Jutel M, Karydas A, Kaye JA, Leszek J, Miller BL, Minthon L, Quinn JF, Rabinovici GD, Robinson WH, Sabbagh MN, So YT, Sparks DL, Tabaton M, Tinklenberg J, Yesavage JA, Tibshirani R, Wyss-Coray T (2007). Classification and prediction of clinical Alzheimer’s diagnosis based on plasma signaling proteins. Nat Med.

[CR65] Roher AE, Esh CL, Kokjohn TA, Castaño EM, Van Vickle GD, Kalback WM, Patton RL, Luehrs DC, Daugs ID, Kuo YM, Emmerling MR, Soares H, Quinn JF, Kaye J, Connor DJ, Silverberg NB, Adler CH, Seward JD, Beach TG, Sabbagh MN (2009). Amyloid beta peptides in human plasma and tissues and their significance for Alzheimer’s disease. Alzheimers Dement.

[CR66] Rosenberg RN, Baskin F, Fosmire JA, Risser R, Adams P, Svetlik D, Honig LS, Cullum CM, Weiner MF (1997). Altered amyloid protein processing in platelets of patients with Alzheimer disease. Arch Neurol.

[CR67] Sevush S, Jy W, Horstman LL, Mao WW, Kolodny L, Ahn YS (1998). Platelet activation in Alzheimer disease. Arch Neurol.

[CR68] Shad KF, Aghazadeh Y, Ahmad S, Kress B (2013). Peripheral markers of Alzheimer’s disease: surveillance of white blood cells. Synapse.

[CR69] Skovronsky DM, Lee VM, Praticò D (2001). Amyloid precursor protein and amyloid beta peptide in human platelets. Role of cyclooxygenase and protein kinase C. J Biol Chem.

[CR70] Smith CC, Betteridge DJ (1997). Reduced platelet serotonin content and release in familial hypercholesterolaemia. Atherosclerosis.

[CR71] Song F, Poljak A, Smythe GA, Sachdev P (2009). Plasma biomarkers for mild cognitive impairment and Alzheimer’s disease. Brain Res Rev.

[CR72] Sparks DL, Scheff SW, Hunsaker JC, Liu H, Landers T, Gross DR (1994). Induction of Alzheimer-like beta-amyloid immunoreactivity in the brains of rabbits with dietary cholesterol. Exp Neurol.

[CR73] Srisawat C, Junnu S, Peerapittayamongkol C, Futrakul A, Soi-ampornkul R, Senanarong V, Praditsuwan R, Assantachai P, Neungton N (2013). The platelet amyloid precursor protein ratio as a diagnostic marker for Alzheimer’s disease in Thai patients. J Clin Neurosci.

[CR74] Stellos K, Panagiota V, Kögel A, Leyhe T, Gawaz M, Laske C (2010). Predictive value of platelet activation for the rate of cognitive decline in Alzheimer’s disease patients. J Cereb Blood Flow Metab.

[CR75] Tang K, Hynan LS, Baskin F, Rosenberg RN (2006). Platelet amyloid precursor protein processing: a bio-marker for Alzheimer’s disease. J Neurol Sci.

[CR76] Tanzi RE, McClatchey AI, Lamperti ED, Villa-Komaroff L, Gusella JF, Neve RL (1988). Protease inhibitor domain encoded by an amyloid protein precursor mRNA associated with Alzheimer’s disease. Nature.

[CR77] Tapiola T, Alafuzoff I, Herukka SK, Parkkinen L, Hartikainen P, Soininen H, Pirttilä T (2009). Cerebrospinal fluid {beta}-amyloid 42 and tau proteins as biomarkers of Alzheimer-type pathologic changes in the brain. Arch Neurol.

[CR78] Trillo L, Das D, Hsieh W, Medina B, Moghadam S, Lin B, Dang V, Sanchez MM, De Miguel Z, Ashford JW, Salehi A (2013). Ascending monoaminergic systems alterations in Alzheimer’s disease. Translating basic science into clinical care. Neurosci Biobehav Rev.

[CR79] Tukiainen E, Wikström J, Kilpeläinen H (1981). Uptake of 5-hydroxytryptamine by blood platelets in Huntington’s chorea and Alzheimer type of presenile dementia. Med Biol.

[CR80] Ullrich C, Pirchl M, Humpel C (2010). Hypercholesterolemia in rats impairs the cholinergic system and leads to memory deficits. Mol Cell Neurosci.

[CR83] Zainaghi IA, Talib LL, Diniz BS, Gattaz WF, Forlenza OV (2012). Reduced platelet amyloid precursor protein ratio (APP ratio) predicts conversion from mild cognitive impairment to Alzheimer’s disease. J Neural Transm.

[CR84] Zetterberg H, Blennow K, Hanse E (2010). Amyloid beta and APP as biomarkers for Alzheimer’s disease. Exp Gerontol.

